# When the Number Matters: Contextualising Australian Reference Values for ^99^ᵐTc‐Pertechnetate Thyroid Uptake

**DOI:** 10.1002/jmrs.70129

**Published:** 2026-09-30

**Authors:** Geoffrey Schembri, Jeremy Hoang

**Affiliations:** ^1^ Department of Nuclear Medicine Royal North Shore Hospital Sydney New South Wales Australia; ^2^ Faculty of Medicine and Health University of Sydney Sydney New South Wales Australia; ^3^ Hornsby Ku‐Ring‐Gai Hospital Sydney New South Wales Australia

**Keywords:** Australia, Graves disease, reference values, sodium pertechnetate Tc 99 m, thyroid gland

## Abstract

Are the historical 1%–4% thyroid uptake reference values still valid in modern practice? This editorial examines why contemporary Australian and international data suggest otherwise and situates the new reference range reported by Wornes (https://doi.org/10.1002/jmrs.70078) and colleagues within the broader international literature. It further identifies the one clinical scenario—distinguishing mild Graves' disease from silent thyroiditis—where quantitative uptake measurement genuinely changes management and argues for a nationally coordinated, prospectively designed re‐derivation study.

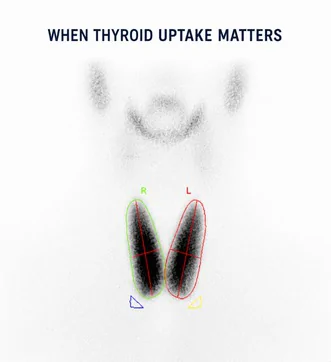

The study by Wornes and colleagues published in this issue addresses a gap that has persisted in Australian nuclear medicine practice for over half a century. Their derivation of a ^99^ᵐTc‐pertechnetate thyroid uptake reference range of 0.4%–2.4% from a consecutive cohort of 151 euthyroid adults at a Victorian quaternary centre is, to the authors' knowledge and our own, the first such dataset published for an Australian population [1].

The reference range of 1%–4% against which Wornes et al. calibrate their findings was first described in Atkins' 1971 review [2]. Both studies used scintillation probe methodology, a fundamentally different acquisition geometry from the low‐energy high‐resolution (LEHR) parallel‐hole planar camera technique employed in contemporary reference range studies. Jin and colleagues described planar camera‐derived ranges of 0.3%–1.9% against probe‐derived ranges of 2.0%–4.7%, a greater than twofold difference [3]. Hence the observed departure of modern camera‐based normal ranges from the historical 1%–4% benchmark likely reflects two distinct phenomena: a genuine population‐level change in dietary iodine status over decades, and a systematic methodological artefact arising from instrument transition.

The attribution of lower contemporary uptake values to dietary iodine shifts is biologically plausible. Australian milk iodine concentrations were historically elevated approaching 600 μg/L in the mid‐1970s (mean 583–593 μg/L in a 1975 survey of New South Wales and Victorian milk) [4]. This was an inadvertent consequence of iodophor‐based sanitisers used in the dairy industry. Progressive substitution of iodophors with chlorine‐based alternatives reduced median milk iodine content in Sydney to approximately 140 μg/L by 2001 [4]. A subsequent national iodine nutrition study found median urinary iodine excretion below the World Health Organisation threshold of 100 μg/L in New South Wales and Victoria, confirming that iodine deficiency had re‐emerged in the population. The policy response of mandatory iodisation of salt in commercial bread production produced measurable improvement in population urinary iodine concentrations in subsequent surveys [5]. Higher dietary iodine availability suppresses thyroid sodium‐iodide symporter (NIS) expression through intrathyroidal autoregulation [6] and it follows that these changes would be expected to produce corresponding shifts in population‐level pertechnetate uptake. However, none of the published reference range studies measured urinary iodine concentration concurrently in their participants and none attempted to stratify uptake values by iodine status. The degree to which the observed departure from historical 1%–4% values reflects iodine status change versus acquisition method transition therefore cannot be determined from existing data.

These caveats notwithstanding, the findings of Wornes et al. are consistent with the international literature. Several directly comparable reference range studies have been published over the past two decades, spanning the United Kingdom, Saudi Arabia, Nepal, Germany and Jordan [7, 8, 9, 10, 11]. Recruitment strategies differ: the United Kingdom and Saudi studies identified euthyroid subjects within dual‐tracer subtraction parathyroid scintigraphy cohorts [7, 8], whereas the Nepalese study prospectively enrolled healthy euthyroid volunteers [9], the German multicentre study drew on euthyroid patients undergoing scintigraphy for thyroid nodules [10], and the Jordanian series derived its normal range from euthyroid patients within a thyrotoxicosis‐workup cohort [11]. This heterogeneity of source population is a source of between‐study variation. The camera‐based 95th percentile upper limits from these studies cluster between 1.6% and 2.4%, with the majority falling below 2.0%. The Wornes upper limit of 2.4% sits at the higher end of this distribution, aligning with the 2024 German study [10]. The inverse age association and the absence of a sex difference are reproduced across most comparator studies [7, 8, 10], lending support to their interpretation as genuine biological signals. This is not universal, however. The Nepalese cohort reported a positive age association [9], although that correlation is weak (*r* = 0.28, ~8% of the variance) and the published scatter indicates it is driven substantially by a small number of high‐value older participants.

Review of the acquisition methodology across the comparator studies reveals substantial heterogeneity. Camera vendors where stated include Mediso (Jordan), Siemens (Nepal) and GE (Wornes et al.); the UK study does not report camera vendor. The Saudi study used a pinhole collimator rather than the LEHR parallel‐hole geometry—a fundamentally different acquisition geometry [8]. Denominator calculations range from phantom sensitivity calibration (UK) to camera‐derived syringe count subtraction (Nepal, Australia) to dose calibrator measurement (Jordan). Administered activities span an approximately 40‐fold range from 18.5 MBq (Jordan) to 740 MBq (Saudi Arabia). Processing algorithms are unreported in most studies. In this context, the convergence of numerical reference ranges is arguably more remarkable than their variation and suggests the derived values are reasonably robust at a population level. Nevertheless, the absolute uptake percentage calculated by any given algorithm is platform‐specific: the Wornes reference range is strictly valid for GE D670/D870 SPECT/CT cameras processed through the Xeleris/Aladdin algorithm and should not be uncritically adopted without local validation. This limitation affects all studies, not just the current study.

Several further limitations deserve acknowledgement beyond those identified by the authors. The parathyroid‐derived recruitment strategy introduces a systematic demographic skew. The cohort is older (median age 62 years) and predominantly female (67.5%), reflecting the epidemiology of primary hyperparathyroidism rather than the broader population presenting for thyroid evaluation. The significant difference in uptake between the 2021 prospective cohort and the 2017–2020 retrospective cohort can be attributed to procedural and workflow factors including the introduction of residual activity measurement. This explanation is reasonable, but it also demonstrates the sensitivity of the calculated uptake percentage to methodological variation.

A further limitation warrants closer scrutiny. Thirty‐five per cent of participants lacked a TSH result within the required window. Rather than excluding these patients, the authors used scan appearance and clinical notes to make a judgement of euthyroid status. This creates a circularity where the scan itself is the outcome being measured. The authors performed a sensitivity analysis restricted to those participants with a confirmed normal TSH and the derived reference range was unchanged. This is reassuring and provides reasonable grounds for accepting the full cohort result but does not entirely eliminate the underlying concern. Future studies should include biochemical euthyroid confirmation in all participants.

The most important question these data implicitly raise is one they do not address: in an era of high‐sensitivity TSH assays, quantitative thyroid autoantibody measurement and colour Doppler ultrasonography, how much additional diagnostic benefit does the quantitative pertechnetate uptake percentage provide?

Pertechnetate is transported into thyroid follicular cells by NIS, a basolateral membrane protein whose expression is driven principally by TSH acting through the TSH receptor [6]. Unlike iodide, pertechnetate is not organified, so the measured uptake reflects NIS activity and follicular cell mass rather than hormonal synthesis [6]. In Graves' disease, TSH receptor antibodies (TRAb) substitute for TSH as the driver of NIS upregulation, producing characteristically elevated uptake. In most other euthyroid individuals, uptake is a direct function of prevailing TSH stimulation, which explains the age‐related decline and the absence of a sex effect [7, 8, 10]. In thyroiditis, near‐absent uptake reflects two converging mechanisms: first, passive release of preformed thyroid hormone suppresses TSH through normal pituitary feedback, removing the principal stimulus for NIS expression [12]; and second, the inflammatory microenvironment is thought to suppress residual NIS transcription through cytokine‐mediated pathways, principally TNF‐α acting via NF‐κB signalling—a mechanism demonstrated in vitro in thyroid follicular cell models. The released hormone is predominantly T4, reflecting the T4:T3 molar ratio of thyroglobulin‐stored hormone, which is reported to range between approximately 14:1 and 20:1. This is in contrast to Graves' disease where active synthesis under chronic TRAb stimulation produces a relatively T3‐enriched secretory pattern [12].

Experienced reporters do not assess thyroid tracer activity in isolation. Salivary gland NIS expression is TSH‐independent [6], and salivary gland activity therefore provides a useful internal reference standard on every pertechnetate scan. This partially substitutes for a numerical threshold in routine reporting and in the majority of presentations this is sufficient.

For most clinical presentations in which thyroid scintigraphy is requested, the pertechnetate thyroid uptake percentage adds little diagnostic value to the scintigraphic appearance. In florid Graves' disease, diffusely increased homogeneous uptake is diagnostic; the uptake percentage confirms what the image already shows. In overt subacute thyroiditis, near‐absent tracer visualisation also makes the diagnosis. The Jordanian experience with 209 patients across all categories of thyroid dysfunction confirms this: one‐third of thyrotoxic patients had uptake values falling within the overlap zone between normal and pathological ranges, and diagnosis in these patients required qualitative image interpretation [11].

There is, however, one well‐defined clinical scenario in which quantitative uptake measurement carries genuine independent diagnostic weight: the distinction between mild or early Graves' disease and painless or silent thyroiditis in a patient with biochemical thyrotoxicosis where the scintigraphic pattern is equivocal. Uchida and colleagues demonstrated that a camera‐based uptake cut‐off of 1.0% discriminated Graves' disease from painless thyroiditis with sensitivity of 96.6% and specificity of 97.1%, and that blinded expert diagnosis based on clinical biochemistry and image pattern alone without the uptake value was inferior [13].

Currie and Iqbal found that visual‐only interpretation without quantitative uptake values reduced pooled physician diagnostic accuracy from 82.3% to 61% [14]. If generalisable, this would constitute a substantive argument for retaining routine quantitative reporting. However, this retrospective cohort included a substantial proportion of subclinical hyperthyroidism and T3 toxicosis, which are states atypical of a standard thyroid scintigraphy referral population, and the reference cut‐off applied reflected acquisition methodology quite different from the contemporary Australian context. The finding merits acknowledgement but does not overturn the position that in routine presentations, pattern‐based visual interpretation remains the primary diagnostic tool.

The practical implication for the current paper is that the clinical scenario in which quantitative uptake carries the most weight‐mild Graves' versus silent thyroiditis‐is precisely the scenario in which the calibration of the normal range is most important. An institution still applying the 1%–4% range derived from probe‐era data from a foreign population [2] risks systematic misclassification in exactly the cases where the quantitative value is most needed. From this perspective, the Wornes study [1] is not merely an academic exercise in local validation it is a prerequisite for the safe and rational use of the one quantitative parameter whose clinical value is supported by evidence.

The broader lesson is one of institutional quality assurance. Reference ranges derived from probe‐era studies in overseas populations have propagated through Australian nuclear medicine practice for 50 years, sustained by inertia rather than evidence. The case for periodic local re‐derivation—particularly following instrument transitions, changes to radiopharmaceutical preparation protocols or shifts in the background dietary iodine environment—is clear and should be reflected in guidance from the peak bodies. Future work in this area would benefit from prospective design, concurrent urinary iodine concentration measurement, mandatory TSH confirmation in all participants, multi‐vendor acquisition protocols and sufficient sample sizes to support age‐stratified interval reporting. The 2024 German multicentre study incorporating TSH‐normalised uptake and urinary iodine concentration represents the current methodological frontier and provides a useful template [10]. Australian and New Zealand centres are well‐placed to collaborate on a study of this design, and the Wornes paper provides both the motivation and the preliminary local data to anchor such an effort.

## Conflicts of Interest

The authors declare no conflicts of interest.

## Data Availability

The authors have nothing to report.
